# Evolutionary Invasion and Escape in the Presence of Deleterious Mutations

**DOI:** 10.1371/journal.pone.0068179

**Published:** 2013-07-17

**Authors:** Claude Loverdo, James O. Lloyd-Smith

**Affiliations:** 1 Department of Ecology and Evolutionary Biology, University of California Los Angeles, Los Angeles, California, United States of America; 2 Fogarty International Center, National Institutes of Health, Bethesda, Maryland, United States of America; Centers for Disease Control and Prevention, United States of America

## Abstract

Replicators such as parasites invading a new host species, species invading a new ecological niche, or cancer cells invading a new tissue often must mutate to adapt to a new environment. It is often argued that a higher mutation rate will favor evolutionary invasion and escape from extinction. However, most mutations are deleterious, and even lethal. We study the probability that the lineage will survive and invade successfully as a function of the mutation rate when both the initial strain and an adaptive mutant strain are threatened by lethal mutations. We show that mutations are beneficial, i.e. a non-zero mutation rate increases survival compared to the limit of no mutations, if in the no-mutation limit the survival probability of the initial strain is smaller than the average survival probability of the strains which are one mutation away. The mutation rate that maximizes survival depends on the characteristics of both the initial strain and the adaptive mutant, but if one strain is closer to the threshold governing survival then its properties will have greater influence. These conclusions are robust for more realistic or mechanistic depictions of the fitness landscapes such as a more detailed viral life history, or non-lethal deleterious mutations.

## Introduction

When a population of replicators faces a sudden change in its environment, often its fitness will decrease so that it has to adapt or face extinction. Examples include when a parasite infects a new host species, when a species is introduced to a new ecological niche, when viruses or bacteria are challenged by an antimicrobial drug administered to their host, or when a cancerous cell invades a new tissue [1]. If adaptive genotypes (i.e. which increase fitness) exist within mutational range, the replicator population has the opportunity to adapt and survive, in a process termed evolutionary escape or evolutionary invasion. Past models of evolutionary invasion and escape have generally ignored mutations that are off the pathway to adaptation [2]–[4], and have concluded that higher mutation rates lead to higher survival probability for the replicator's lineage, i.e. higher invasion or escape probability. The same conclusion is often implied in the empirical literature. For instance, it is often said that RNA viruses are the leading cause of emerging infectious diseases because their high mutation rate enables them to adapt more easily to new host species [5]–[9].

However, it is known that most mutations are deleterious or even lethal. In the case of viruses, for example, site-directed mutagenesis experiments have shown that 20 to 40% of point mutations in various viruses are lethal [10]. Mutations can be lethal because they introduce a stop codon, disrupt the production of a crucial protein, affect key reactive sites of proteins, or disrupt the interaction of the genome itself with other proteins. The probability that another mutation can compensate for such changes is very small, so the presence of any non-zero number of such mutations typically makes the virus non-viable. Indeed, the mechanism of action of some antiviral drugs is thought to be lethal mutagenesis [11], i.e. increase of the mutation rate to levels where the probability that a new genome has at least one lethal mutation is high enough to threaten the survival of the viral population [12]. In numerous analyses of this phenomenon [12]–[14], any increase in the mutation rate is assumed to endanger the initially fit virus. Looking more broadly, deleterious mutations are a burden for all replicators, not only viruses [15]. The observed mutation rate often seems to result from a trade-off between the cost of deleterious mutations and the cost of achieving high-fidelity replication [16]–[19].

If a replicator does not mutate at all, it never adapts, and then cannot survive environmental changes. But if a replicator mutates too often, it also carries a deleterious mutational load. The concept of mutations as a double-edged sword has been explored in many situations. For example, Bull studied the mean number of adaptive mutants produced by a single episode of mutagenesis [20], while Iranzo et al. calculated the mean growth rate of a pathogen population exposed to a combination of a drug reducing growth and another drug increasing the mutation rate [21]. There is an extensive literature on adaptation rates (i.e. fixation rates of adaptive mutations) in a population of constant size [22]–[24], or with a given demographic trajectory [25]. However, in the case of evolutionary invasion and escape, the most important quantity is the probability of survival of a replicator's lineage, because if the lineage survives the population will grow until limited by other factors (such as resource availability). Maximizing the survival probability of a replicator's lineage is different from maximizing the adaptation rate in a population of fixed size. In both cases, an important quantity is the probability to generate mutants bearing an adaptive mutation but no deleterious mutations. But in the former case, deleterious mutations decrease both the survival probability of a lineage of replicators of the initial type when there is no adaptive mutant, and the probability of survival of a lineage initiated by an adaptive mutant, which places additional constraints on the mutation rate.

To our knowledge, only two studies have looked at the probability of survival of a replicator's lineage when both deleterious and adaptive strains are within mutational range. Eshel [26] proved that a finite mutation rate maximizes the survival probability of a replicator's lineage when an initial unfit strain needs to mutate to a fitter strain to survive, but this fitter strain is threatened by lethal mutations, so that it cannot survive if the mutation rate is too high. Alexander & Day [27] studied two scenarios. First, when mutations to the initial strain are either adaptive or lethal, and adaptive strains are assumed not to mutate at all, then increasing mutation rate leads to monotonic increase or decrease in survival probability depending on the fitness of the initial strain. Second, when two strains of different fitness are linked by mutations in both directions, there is a parameter regime where an intermediate mutation rate maximizes survival.

We provide a more complete and unified analysis of the influence of deleterious and lethal mutations on the phenomenon of evolutionary invasion and escape. We develop and analyze a general stochastic model for the survival probability of a replicator lineage that begins with an arbitrary fitness, and can acquire mutations that are adaptive, deleterious, or lethal. We derive simple, biologically intuitive rules to delineate when mutations are beneficial (i.e. when a positive mutation rate leads to greater survival probability than the limit of no mutations), and in this regime, we calculate the optimal mutation rate (i.e. the mutation rate maximizing the survival probability of the replicator lineage for the environmental change being studied). This model can encompass the earlier results of Eshel[26] and Alexander & Day [27] as special cases, and places their findings in the context of broader conclusions about the impact of deleterious mutations on evolutionary escape. We then extend our general model to incorporate greater realism, considering more complex genotype spaces and fitness landscapes, and analyze a particular scenario based on a mechanistic model for within-host viral dynamics. We highlight the robust conclusions that apply for all scenarios considered, and discuss the implications for viral emergence and the evolution of mutation rates.

## Methods and Results

### General model: evolutionary invasion with adaptive and lethal mutations

#### Model framework

We study asexual replicators, such as cancerous cells, viruses with negligible recombination, or bacteria with negligible horizontal transfer. For sexual replicators the effects of deleterious mutations are mitigated by recombination and exhibit a very different sensitivity to the mutation rate [28].

We consider whether an initial population of replicators in a novel environment leads to establishment of a successful population or goes extinct. Situations where replicators jump to a new environment often involve only a few explorers, or else abrupt environmental change can sharply decrease the size of an extant population. Also any population is most likely to go extinct when there are the fewest replicators present. For all these reasons, we focus on the dynamics of a replicator population that begins at low abundance. When there are few replicators, the interactions between them are limited, and depletion of resources is negligible. Hence for calculating the survival probability we assume that the demographic fates of replicators are independent. Consequently, we use a branching process framework [29], as is standard in the evolutionary escape literature [1], [2], [27]. We study the case of one initial replicator, but given the assumption of independence, the dynamics for 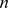 initial replicators can be deduced directly from the dynamics of each of them. In particular, the probability of extinction of the entire founding population is the product of the extinction probabilities for each replicator considered alone.

To analyze the branching processes, we use generating functions which gather the information on the probabilities 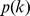 that a single replicator produces 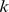 replicators after the next event in the system: 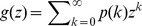. Standard branching process theory implies that the probability of eventual extinction 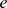 is given by the smallest positive solution to 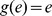 (and the survival probability is 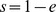) [29]. This can be extended to multitype branching processes, with the generating map 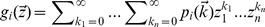 where 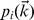 is the probability that one replicator of strain 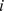 produces a set of 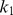 replicators of strain 1, 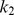 replicators of strain 2, etc [29]. We assume that mutations occur upon replication.

**Figure 1:**
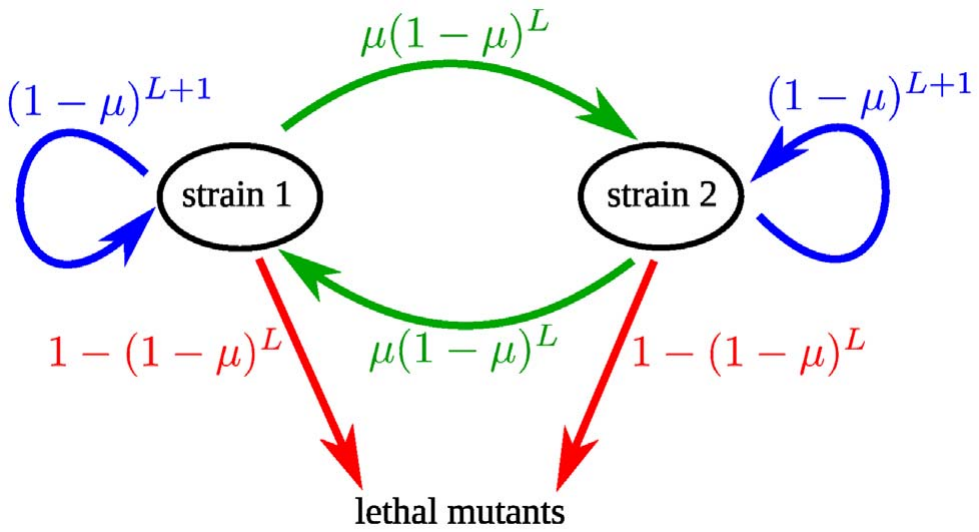
Mutational map. A mutation at any of the 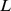 lethal sites of the genome makes the strain non-viable, and a mutation at the one adaptive site increases the fitness. The mutation rate 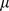 is the same for all sites.

A mutation in the genome may change fitness. In the context of adaptation to new conditions, mutations with a significant effect on replicator fitness (e.g. those that modify a reactive site on a protein for instance) can be distinguished from mutations with small effect (e.g. those that marginally alter the thermodynamic stability of a protein) [30]. Likewise, deleterious mutations can range from lethal mutations with dramatic deleterious effects on replicator fitness (e.g. a stop codon in an essential gene) to much milder effects [10], [31]. Approximating small-effect mutations as neutral mutations, we consider an idealized fitness landscape for a genome consisting of 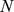 sites, where 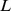 sites are lethal, i.e. the strain is non-viable if any of these sites is mutated, one site allows for adaptive mutations, and the 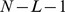 remaining sites are neutral (figure 1). Each site represents a nucleotide, so a mutation is a nucleotide substitution, though the model framework could be modified to address allele changes across genes. We assume that the mutation rate is the same for all sites. The parameter 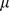 is the probability of mutation at each site, given birth of a new replicator. We use the term “mutation rate” for consistency with the biological literature. For a real system, mutation rates can be different for different sites or for back mutations, but if a change in the mean mutation rate affects all rates proportionally (e.g. a more error-prone enzyme for replication or proof-reading), this would not affect our qualitative conclusions.

### Analysis and results

#### Generating functions

We consider a replicator of strain 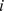 (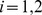) which duplicates at a rate 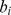 per unit time and dies at a rate 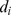 per unit time. The basic reproductive number, i.e. the mean number of direct descendants of this replicator, is 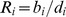. We construct the generating function by considering the probability of each type of event and the resulting number of replicators of each strain. The next event is death with probability 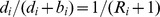, which leads to a term in 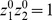. The next event is replication with probability 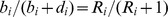, which leads to a term in 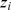 (for the parent replicator) 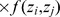 (for the offspring replicator, which can be mutated as explained in figure 1: 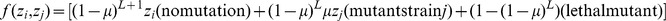). Thus the generating function starting from one replicator of strain 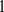 is:
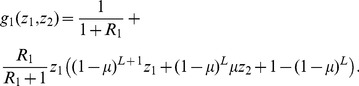
(1)


The extinction probabilities 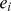 starting from one replicator of strain 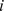 are solutions of the system 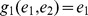 and 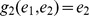.

#### Influence of the mutation rate on survival

**Figure 2:**
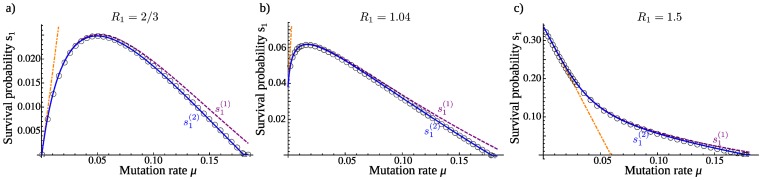
Survival probability of the replicator's lineage starting from one replicator of strain 1 as a function of the mutation rate, for three values of the initial strain fitness 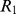. Exact solution (black circles), approximation for small 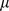 (orange dot-dashed-line), and iterative approximations 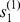 (purple dashed line) and 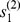 (blue solid line). 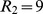, 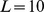.

Solving this system numerically shows the dependence of survival probability on mutation rate (black circles in figure 2). All three panels show the scenario where there is a single site where mutation is adaptive (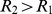) and 10 sites where mutation is lethal (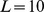). For small enough values of 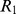 (figure 2 a,b), increasing the mutation rate from a low level increases survival, as more adaptive mutants are produced. But there is a finite mutation rate at which the survival probability is maximized, because at higher mutation rates the fitness burden of lethal mutations is greater. When the initial fitness of the introduced strain is higher (figure 2c), this latter effect dominates, and any amount of mutation decreases the survival probability of the replicator lineage. Thus even when a neighboring genotype is substantially fitter, an increase in the mutation rate can be disadvantageous. To generalize this finding and derive biological insights about its determinants, we analyze the survival probabilities further for several basic scenarios.

#### Regime of beneficial mutations

To investigate whether mutations are beneficial at all in a given scenario, we study whether a small amount of mutation leads to more survival than no mutations, i.e. whether the initial slope of the survival probability as a function of the mutation rate is positive (orange dot-dashed lines on figure 2). We introduce a new quantity, the survival probability without mutations, which is 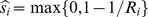. We can then derive approximations for the survival probability starting from one replicator of strain 1, in the limit of low mutation rate (see Appendix S1.1 in file S1). If 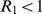 (hereafter referred to as an “unfit” strain), the lineage dies out with certainty in the absence of mutations, and:
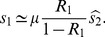
(2)


If 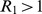 (hereafter referred to as a “fit” strain), the lineage can survive without mutation, and:
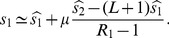
(3)


If the coefficient of 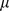 is positive in these expressions, mutations are beneficial. If the initial strain is unfit (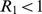), mutations are always beneficial, because they are necessary to have any chance to avoid extinction. If the initial strain is fit (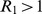), the presence of lethal mutants means that mutations are beneficial when the adaptive strain is much fitter, more precisely when 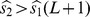.

This result can be generalized to the following simple rule (appendix S1.1 in file S1): calculating the survival probabilities in the absence of mutations, if the survival probability averaged over the immediate mutational neighbors is larger than the survival probability of the initial strain, then mutations are beneficial. Immediate mutational neighbors are strains one mutation away from the initial strain, which in the particular case above are 1 neighbor of survival probability 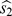 and 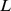 neighbors of survival probability 0. This is a sufficient condition to prove that mutations are beneficial. But it is not a necessary condition, as we will see when several mutations are needed to reach a fitter strain.

#### Optimal mutation rate

When mutations are beneficial, there is a finite mutation rate that maximizes the probability of survival for a given environmental change scenario, which we refer to as the optimal mutation rate, 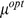. To investigate how this optimum depends on the parameters of the model, we build approximations for the survival probabilities (figure 2). We define 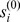 as the survival probability that accounts for lethal mutations only. A first approximation step 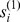 is to neglect the back mutations from strain 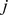 to strain 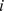, i.e. writing the survival probability starting from a replicator of strain 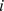 as a function of the survival probability starting from a replicator of strain *j*, and taking 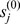 as the value for the latter. The next step is 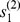, i.e. 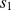 calculated using 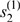 as a value for 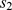. We make further approximations based on these expressions (appendix S1 in file S1).

These approximations do not lead to a simple explicit expression for 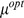, but they do give analytical insights about the factors that influence the optimal mutation rate when the initial strain is unfit (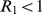). When the number of lethal mutants is large (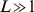), the mutation rate that maximizes survival is proportional to 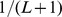: as expected, the greater the frequency of lethal mutations, the lower the optimal mutation rate. Interestingly, in the limit 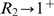 (the mutant barely survives) 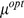 does not depend anymore on 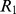, and in the limit 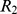 large (the mutant is very fit) 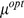 does not depend on 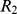. Thus the optimal mutation rate seems to depend only on the parameters of the strain that is closer to the threshold value 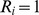 governing survival, for which the fine-tuning of the mutation rate 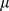 will have the largest impact on survival.

### Towards more realistic fitness landscapes

#### Varying the numbers of lethal mutations

We have assumed that the risk of lethal mutations is the same for both strains. However in real systems there may be epistatic interactions such that strains have different robustness. Furthermore, from our first analysis we cannot conclude whether the results depend on the lethal mutations threatening the initial or the mutant strains. To explore this, we study a model that has two strains of differing fitness, as in Figure 1, where the initial strain is endangered by 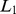 lethal mutations and the adaptive strain is endangered by 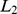 lethal mutations.

Once again we determine the regime of beneficial mutations by considering the low mutation rate limit. In this limit, the survival probability of strain 1 depends on the characteristics of strain 2 only via 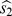, and thus it is independent of 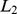. Consequently, the criterion for mutations to be beneficial (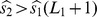) depends only on 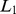, not on 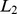. If the initial strain is not endangered with too many lethal mutations, mutations increase survival.

**Figure 3:**
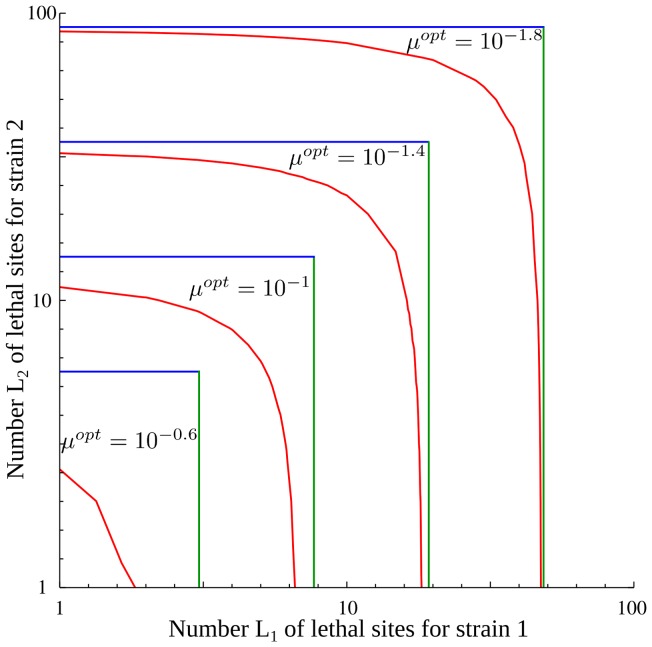
The optimal mutation rate as a function of *L*_1_ and 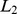. Red lines: exact numerical solution showing combinations of 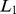 and 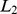 that give the indicated value of 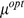, for the other parameters as given below. Green vertical lines: approximation depending on 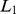 and 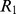 only. Blue horizontal lines: approximation depending on 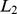 and 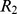 only. 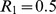, 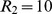.

The optimal mutation rate depends on 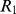, 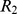, 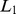 and 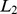. However, by refining the initial iterative approximation in the regime 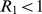 (appendix S2 in file S1), we find that in the limit where both 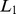 and 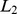 are large, but one is much larger than the other, only the parameters of the strain threatened with more lethal mutations matter (figure 3). The same phenomenon holds qualitatively for smaller values of 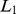 and 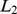. Thus to optimize the mutation rate, only the less robust strain has to be taken into account.

#### Two mutational steps needed to reach an adaptive strain

**Figure 4:**
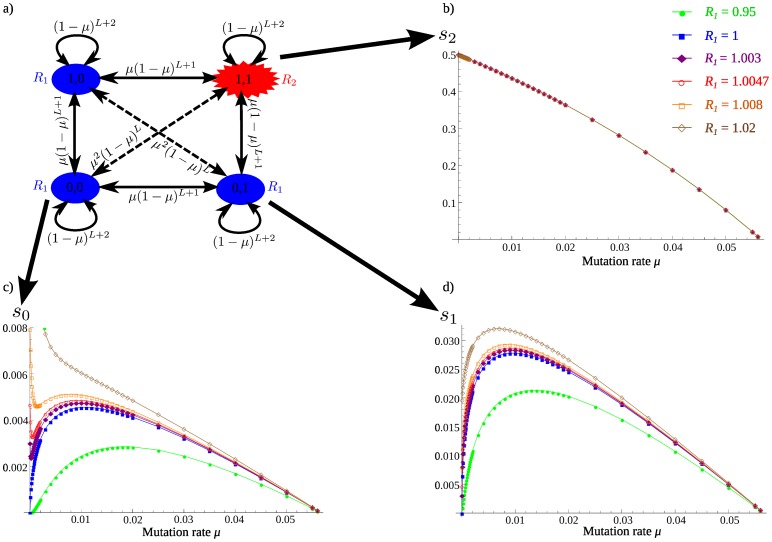
Survival probabilities as a function of the mutation rate when two mutations are needed to increase fitness. Panel (a) represents the mutational map. There are 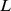 lethal sites on the genome, and 2 adaptive sites. The initial strain (0,0)and the strains with a mutation at one of the adaptive sites 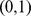 and 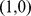 have reproductive number 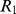. The strain bearing both mutations 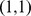 has a reproductive number 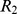. Survival probabilities starting from a replicator of strain 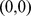 (c), 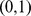 or 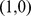 (d) and 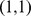 (b) are represented as a function of the mutation rate 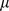. 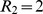, 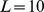.

Often a significant increase in fitness requires more than one mutation [32], [33]. How does this affect our conclusions? Here we study a simple model where two mutations are needed to obtain a higher reproductive number 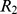, while the non-mutant and the one-mutation strains have the same reproductive number *R*_1_, with 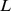 possible lethal mutations for all strains (see figure 4a and appendix S3 in file S1). We denote the different strains by their mutational states at the two sites, from the initial strain (0,0) to the double mutant 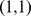.

We consider how increasing the mutation rate affects the survival probability for a population starting from a single replicator of each genotype. Mutations are always a burden for the fittest replicator 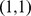, and its survival is almost not influenced by the reproductive number of the neighboring strains as long as their survival probability is much smaller (figure 4b). The survival probability starting from a single-mutant replicator (1,0) or 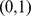 is very similar to the survival probability when only one mutation is required for adaptation (figure 4d; and figure S3 of appendix S3 in file S1). Starting from a replicator with no mutations 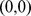, the patterns are more complex (figure 4c). If 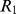 is not too large, there is a local maximum in survival probability for a mutation rate 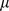 slightly larger than the 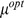 of the single mutant. This arises because there is potential to reach the fitter (1,1) genotype, but the initial strain needs more mutations than the single mutant so its optimal mutation rate is higher. For very low mutation rates, however, there is a negligible chance of reaching the adaptive 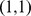 genotype, so if the initial strain is fit (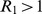), there is a local maximum at 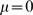. This local maximum is the global optimum when 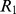 is large enough, since the potential to reach the 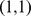 genotype is outweighed by the cost of lethal mutations, but as 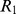 decreases the global optimum switches to the non-zero 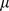 maximum corresponding to the strategy of adaptation. This demonstrates that our earlier criterion for mutations to be beneficial was not necessary but sufficient. If the slope of the survival probability at 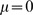 is positive, mutations are certainly beneficial, like before; but if the slope is negative, mutations may still be beneficial at some higher mutation rate.

#### Deleterious mutations

**Figure 5:**
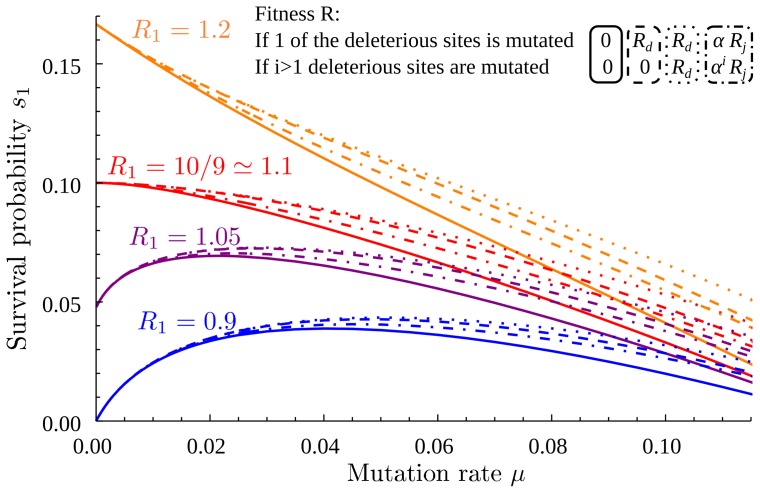
Survival probability as a function of mutation rate, for four different models of deleterious fitness effects. In all cases the model follows the broad scheme laid out in Figure 1, with an initial strain (reproductive number *R*_1_) and a fitter strain (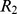) both threatened by 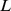 deleterious mutations. Solid lines: our general model with 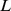 lethal sites; if one of these sites is mutated, 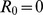. Dashed lines: fitness is 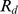 if one of the deleterious sites is mutated, and *R*_0_ = 0 (lethal) if 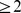 deleterious sites are mutated. Dotted lines: fitness is 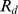 if any (non-zero) number of deleterious sites is mutated. Dot-dashed lines: fitness is , with 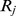 dependent on the allele at the adaptive site and 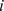 the number of mutated deleterious sites. 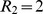, 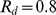, 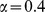, 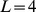. Numeric results using equations detailed in appendix S4 in file S1.

Our analysis so far has assumed that deleterious mutations are all lethal, but of course fitness can decrease without going to zero [10], [15], [31]. We investigated several alternative fitness landscapes with non-lethal deleterious mutants, and found that the outcomes are very similar to our previous results (figure 5). In the limit of low mutation the results are identical, because the initial slope of the survival probability depends on the survival probabilities of mutational neighbors in the absence of mutations, so any type of deleterious mutant with 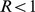 leads to the same ultimate result of extinction. For larger mutation rates, deleterious rather than lethal neighbors lead to moderately higher values of the survival probability and the optimal mutation rate. The fitness of deleterious double mutants has very little influence because more than one mutation is needed to reach them. Overall, what matters most are the immediate mutational neighbors, and deleterious mutations pushing the reproductive number below one act very similarly to lethal mutations, at least at low mutation rates, because they are very likely to be evolutionary dead-ends.

#### Application to within-host viral dynamics

Our replicator model is very general, and may need to be adapted to apply to specific systems. As an example, if we describe the dynamics of a virus within a host, a virion may have a very low probability 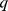 to successfully infect a cell, but when it succeeds, the number 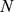 of released virions can be large, up to at least 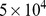 [34]. The basic reproductive number is *R* = *qN*. When many cells are infected, fluctuations will average out and 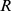 is the dominant parameter describing viral population growth. In the beginning of the infectious process, however, numbers of virions are often low [35], [36] and viral growth is fundamentally stochastic so 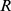 alone may be insufficient to describe the dynamics, as emphasized by Pearson et al. in a non-evolutionary context [37].

We assume that a virion of strain 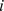 successfully infects a cell with probability *q_i_*, and that this cell has a fixed death rate 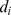 and a fixed rate of production of new virions 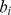, leading to a geometric distribution of the number of new virions produced by this cell of mean 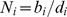. For many common viral life histories, each new virion produced by a cell may bear mutations independently of the others [38], as in the simple model above.

It appears that this description adds two more parameters to our replicator model. However, it can be shown that 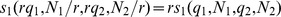 (appendix S5 in file S1), i.e. if we keep the reproductive numbers constant and multiply both probabilities of cell infection 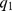 and 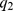 by the same factor 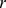, the survival probability is also multiplied by 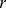. Thus the value of the survival probability changes, but not its dependence on the mutation rate. So when studying the dependence of the survival probability on the mutation rate, the relevant parameters are 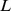, 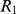 and 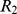, as above, plus one additional parameter, 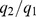, which describes how much more efficiently the mutant strain infects cells compared to the initial strain.

As in the general model, mutations are beneficial when 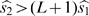. The survival probability of a strain in the absence of mutations is 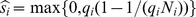 [37], so mutations are beneficial if 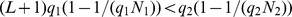. For fixed values of 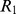 and 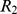, larger ratios 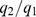 lead to larger ranges of reproductive numbers where mutations are beneficial. That is, if the fitness increase is due predominantly to more efficient infection of new cells, then mutations are more likely to be beneficial.

In principle, there could be situations where a mutation that dramatically increases the number of virions produced by an infected cell (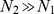) comes at the expense of the probability to infect a cell (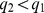). If 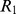 is large enough, such a mutant strain has a higher average growth rate (proportional to 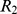), but counter-intuitively a lower survival probability than the initial strain because of increased variance in the number of offspring virions produced [37].

**Figure 6:**
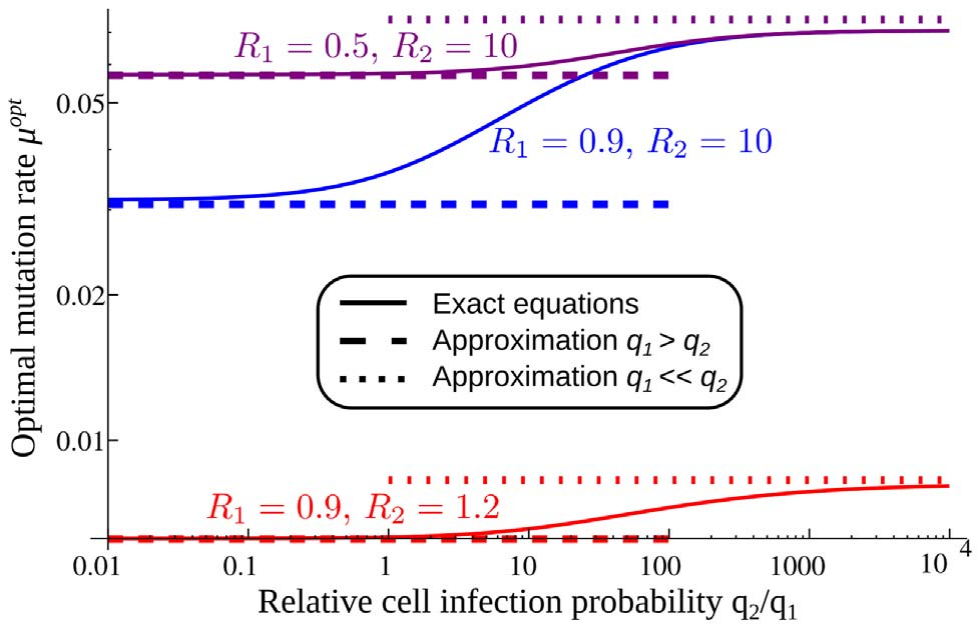
Optimal mutation rate as a function of 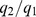
 **for a more realistic model of within-host viral dynamics. ** Numerical result from the exact equations (solid lines), and approximations for 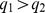 (dashed lines) and 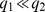 (dotted lines). 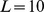.

When the initial strain needs to mutate to survive (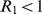 and 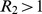), there are two regimes for the optimal mutation rate (figure 6, appendix S5 in file S1). If 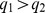, when 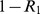 is not too small, the survival probability is the same as for the general model, except for a factor of 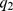, leading to the same dependence on the mutation rate and the same 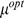. In the regime 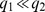, an approximation for small 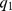 leads to an optimal mutation rate 
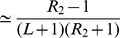
 which depends on 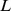 and 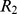 only. Thus the details of the life history of the virus (via the ratio 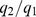) define two regimes, but for each regime 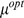 depends on the overall reproductive number 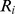 only and not on 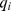 and 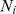 independently.

## Discussion and Conclusion

Replicators facing the threat of extinction because of an environmental change may survive thanks to adaptive mutations. Most models of evolutionary invasion and escape have focused on adaptive mutation pathways only and have concluded that higher mutation rates lead to higher probability that a replicator population will survive an environmental change [2]–[4]. However, empirical evidence shows that most mutations are deleterious, and often they are lethal. When both adaptive and deleterious mutants are considered, an intermediate mutation rate usually maximizes the probability of survival, and in some cases the conclusion is even reversed so that higher mutation rates always lead to lower survival probability, even if an adaptive mutant exists.

We have investigated the conditions under which mutations are beneficial, i.e. when some non-zero level of mutation increases the survival probability compared to the limit of no mutations. A sufficient criterion for mutations to be beneficial is when, in the limit of no mutations, the average survival probability of the mutational neighbors is larger than the survival probability of the initial strain. However, this condition is not necessary: if an adaptive mutant is more than one mutational step away, its influence may be felt at higher mutation rates only. In this case a very low rate of mutations may be detrimental to survival, but the maximum survival probability may be obtained for a larger finite value of the mutation rate.

The optimal mutation rate is defined for our analysis as the mutation rate maximizing the survival probability of a replicator's lineage facing a given environmental change. We first studied a general model with one adaptive mutant and lethal mutations threatening both the initial strain and this adaptive mutant. The optimal mutation rate depends on the characteristics of both the initial strain and the adaptive mutant, but if one of the strains is threatened by more lethal mutations or if it is closer to the survival threshold, the optimal mutation rate depends most strongly on the parameters of this strain. More realistic depictions of the fitness landscape – such as a more mechanistic fitness model for viral infections, or deleterious instead of lethal mutations – do not qualitatively change these results.

Gavrilets has studied such a “holey” fitness landscape comprising both fitness peaks and fitness “holes”, but focused on the distribution of a fixed-size population on such a landscape [39]. Other approaches from population genetics have been used to study the interplay between mutation rate, adaptive and deleterious mutations when population size is fixed and hence they do not consider the risk of extinction [22]–[24]. In the case of evolutionary invasion and escape, the number of replicators is initially small, and not limited by resources, and the question of interest is whether the initial replicators' lineages survive or not. Maximizing the survival of a replicator's lineage is different from maximizing the rate of adaptation (i.e. fixation of adaptive mutants) in a population of a given size. For instance, if the initial strain is fit enough and there are many deleterious mutations, the mutation rate that maximizes survival can be zero, whereas the mutation rate maximizing the adaptation rate is always strictly positive. Some previous models have addressed the demographic dynamics of a population, but have deterministically tracked the expected number of replicators. For instance, Iranzo et al. study the mean growth rate when both mutagenic and inhibitor drugs are applied to a viral population [21]. If in the long term the expected number of replicators goes to zero, extinction is certain. Else, there is still some non-zero probability of extinction, but a stochastic model is needed to calculate it, and it can be high in the case of a small initial population.

To the best of our knowledge, only two studies have presented a stochastic model of evolutionary escape where the dependence of the survival probability on the mutation rate is analyzed in the presence of both deleterious and adaptive mutations [26], [27]. Other studies have considered deleterious mutations in the context of fitness valleys, but these have always been part of mutational paths leading to the only strains with 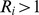, so higher mutation rates are always preferable. In Eshel [26], an unfit strain (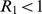) can mutate to a fit strain (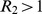) at a rate 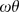, with lethal mutations at a rate 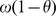 for the unfit strain and 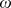 for the fit strain. The initial strain cannot survive without mutations, so the optimal mutation rate is strictly positive. But if 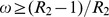, the fit strain will go extinct with certainty, so the optimal mutation rate is bounded below this value. Alexander and Day [27] explored two regimes: one where an unfit strain 1 mutates to a fitter strain 2 at rate 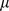, and strain 2 mutates back to strain 1 at rate 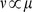 (when 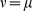 this is equivalent to our general model with 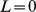); and another where an initial strain 1 mutates irreversibly to 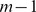 strains, one of which is fitter, and the others are lethal (almost equivalent to our model with 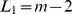 and 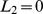, but without back mutations). In the former regime, they observed circumstances where an intermediate level of mutation maximizes survival. In the latter, they showed that despite the existence of an adaptive mutant, mutations can decrease survival if the initial strain is fit enough. Our analysis builds on these results, placing them in a general context and extending them subtantially. We have derived rules that govern when mutations are beneficial and what factors influence optimal mutation rates on more general fitness landscapes, and we have considered the application to viral life histories.

In light of these findings, we return to the question of why so many emerging infectious diseases are RNA viruses. Our analysis has shown that their extremely rapid mutation rates are not necessarily a beneficial trait even if evolutionary adaptation is needed to avoid extinction in the new host species. It is possible that the mutation rates exhibited naturally by RNA viruses, while high, are not so high that they cause survival probabilities to decline markedly. This is difficult to judge in general, because even in our simplified model a quantitative estimate of survival probability requires, at minimum, knowledge of the fitnesses of different genotypes and the frequency of deleterious mutations. It is also possible that RNA viruses are common emerging infections for reasons unrelated to their mutation rate, for instance if there is a larger pool of candidate RNA viruses circulating in animal reservoirs to which human populations are exposed (though see [7]). A high mutation rate is not universally beneficial for emergence and circumspection is needed in invoking it as an explanation for the apparent propensity of RNA viruses to jump host species or otherwise expand their range.

Finally, we place our findings in the context of research on the evolution of mutation rates. Under stable conditions the mutation rate is expected to be small [40], only limited by the cost of reducing replication errors [16]–[18], [41]. However, replicators often face successive environmental changes, as when pathogen or cancer cell lineages have to repeatedly invade new tissue compartments or escape from the adaptive immune system. If the mutation rate can evolve at the same pace or faster than the environmental changes, then low mutations rates are selected when the environment is stable. When the environment changes, the few mutants with a high mutation rate will produce adaptive mutations faster, and will hitch-hike to high frequency with these mutations, but will decline in frequency when the environment stabilizes [42]. Our model shows that even when the environment changes, very high mutation rates are detrimental, so intermediate mutators are more likely to hitch-hike. If the mutation rate evolves on time scales longer than the time scale of environmental change, then one mutation rate can be selected for, as a trade-off between adaptive mutations and the deleterious load. Numerous studies have explored the evolvability of the mutation rate [24], [28], [43]–[45], but they have not integrated the risk of extinction following environmental changes. There are situations in which the survival probability may be the crucial parameter. An example is a parasite in a host, which when it escapes the immune system can grow until limited by resources, or by the next adaptation of the immune system. The survival probability is directly related to the length of infection, which is crucial for transmission, and hence for the parasite's fitness at the scale of the host population. If there are several environmental changes (see appendix S6 in file S1 for a more detailed discussion), steps with the lowest survival probability will matter most, and will select for a mutation rate close to the optimal mutation rate we have calculated for one step (with the strain most adapted to the previous environment as the initial replicator). To explore this situation in greater depth, our results would need to be corrected in two ways: a higher mutation rate may lower the fitness of the population in the previous environment and thus decrease the number of replicators passed to the next environment; but a higher mutation rate also increases the number of pre-existing mutants that are adaptive for the next environment. Future work should integrate these new results into a larger framework dealing with the evolution of the mutation rate and the frequency of environmental change.

## Supporting Information

File S1 **Calculations are detailed in the supporting pdf file, organized in the sections: • S1 General model. • S2 Different numbers of lethal mutations. • S3 Two steps towards a fitter mutant. • S4 Deleterious mutations. • S5 Within-host viral dynamics. • S6 Repetitively changing environment.**
(PDF)

File S2 **For some calculations and for the figures, Mathematica was used, as shown in the supporting Mathematica file.**
(PDF)

## References

[1] Iwasa Y, Michor F, Nowak MA (2004) Evolutionary dynamics of invasion and escape. J. Theor. Biol. 226: 205–214. 10.1016/j.jtbi.2003.08.014 14643190

[2] Antia R, Regoes RR, Koella JC, Bergstrom CT (2003) The role of evolution in the emergence of infectious diseases. Nature 426: 658–661. 14668863 10.1038/nature02104 PMC7095141

[3] André JB, Day T (2005) The Effect of Disease Life History on the Evolutionary Emergence of Novel Pathogens. Proc. R. Soc. B 272: 1949–1956. 10.1098/rspb.2005.3170 PMC1559879 16191602

[4] Orr H, Unckless R (2008) Population extinction and the genetics of adaptation. Am. Nat. 172: 160–9. 10.1086/589460 18662122

[5] Woolhouse MEJ, Haydon DT, Antia R (2005) Emerging pathogens: the epidemiology and evolution of species jumps. Trends Ecol. Evol. 20: 238–44. 10.1016/j.tree.2005.02.009 PMC7119200 16701375

[6] Shackelton LA, Parrish CR, Truyen U, Holmes EC (2005) High rate of viral evolution associated with the emergence of carnivore parvovirus. Proc. Natl. Acad. Sci. USA 102: 379–84. 10.1073/pnas.0406765102 PMC544290 15626758

[7] Cleaveland S, Haydon DT, Taylor L (2007) Overviews of pathogen emergence: which pathogens emerge, when and why? Curr. Top. Microbiol. Immunol. 315: 85–111. 10.1007/978-3-540-70962-6_5 PMC7122528 17848062

[8] Holmes EC, Drummond AJ (2007) The evolutionary genetics of viral emergence. Curr. Top. Microbiol. Immunol. 315: 51–66. 10.1007/978-3-540-70962-6_3 PMC7120214 17848060

[9] Borderia A, Stapleford K, Vignuzzi M (2011) RNA virus population diversity: implications for inter-species transmission. Curr. Opin. Virol. 1: 1–6. 10.1016/j.coviro.2011.09.012 22440922

[10] Sanjuán R (2010) Mutational fitness effects in RNA and single-stranded DNA viruses: common patterns revealed by site-directed mutagenesis studies. Philos. Trans. R. Soc. Lond. B 365: 1975–82. 10.1098/rstb.2010.0063 PMC2880115 20478892

[11] Anderson J, Daifuku R, Loeb L (2004) Viral error catastrophe by mutagenic nucleosides. Annu. Rev. Microbiol. 58: 183–205. 10.1146/annurev.micro.58.030603.123649 15487935

[12] Bull JJ, Sanjuán R, Wilke CO (2007) Theory of lethal mutagenesis for viruses. J. Virol. 81: 2930–9. 10.1128/JVI.01624-06 PMC1865999 17202214

[13] Manrubia SC, Domingo E, Lázaro E (2010) Pathways to extinction: beyond the error threshold. Philos. Trans. R. Soc. Lond. B 365: 1943–52. 10.1098/rstb.2010.0076 PMC2880120 20478889

[14] Martin G, Gandon S (2010) Lethal mutagenesis and evolutionary epidemiology. Philos. Trans. R. Soc. Lond. B 365: 1953–63. 10.1098/rstb.2010.0058 PMC2880112 20478890

[15] Lynch M, Blanchard J, Houle D, Kibota T, Schultz S, et al (1999) Spontaneous deleterious mutation. Evolution 53: 645–663. 28565627 10.1111/j.1558-5646.1999.tb05361.x

[16] Sniegowski PD, Gerrish PJ, Johnson T, Shaver A (2000) The evolution of mutation rates: separating causes from consequences. BioEssays 22: 1057–66. 11084621 10.1002/1521-1878(200012)22:12<1057::AID-BIES3>3.0.CO;2-W

[17] Furió V, Moya A, Sanjuán R (2005) The cost of replication fidelity in an RNA virus. Proc. Natl. Acad. Sci. USA 102: 10233–7. 10.1073/pnas.0501062102 PMC1177365 16006529

[18] Thébaud G, Chadoeuf J, Morelli MJ, McCauley JW, Haydon DT (2010) The relationship between mutation frequency and replication strategy in positive-sense single-stranded RNA viruses. Proc. R. Soc. B 277: 809–17. 10.1098/rspb.2009.1247 PMC2842737 19906671

[19] Lynch M (2010) Evolution of the mutation rate. Trends Genet. 26: 345–352. 10.1016/j.tig.2010.05.003 PMC2910838 20594608

[20] Bull JJ (2008) The optimal burst of mutation to create a phenotype. J. Theor. Biol. 254: 667–73. 10.1016/j.jtbi.2008.06.006 PMC2637111 18619470

[21] Iranzo J, Perales C, Domingo E, Manrubia SC (2011) Tempo and mode of inhibitor mutagen antiviral therapies : A multidisciplinary approach. Proc. Natl. Acad. Sci. USA 108: 16008–16013. 10.1073/pnas.1110489108 PMC3179121 21911373

[22] Orr HA (2000) The rate of adaptation in asexuals. Genetics 155: 961–8. 10835413 10.1093/genetics/155.2.961 PMC1461099

[23] Johnson T, Barton NH (2002) The effect of deleterious alleles on adaptation in asexual populations. Genetics 162: 395–411. 12242249 10.1093/genetics/162.1.395 PMC1462245

[24] André JB, Godelle B (2006) The evolution of mutation rate in finite asexual populations. Genetics 172: 611–26. 16157667 10.1534/genetics.105.046680 PMC1456187

[25] Campos PRA, Wahl LM (2010) The adaptation rate of asexuals: deleterious mutations, clonal interference and population bottlenecks. Evolution 64: 1973–83. 20199567 10.1111/j.1558-5646.2010.00981.x

[26] Eshel I (1973) Clone-selection and optimal rates of mutation. J. Appl. Probab. 10: 728–738.

[27] Alexander HK, Day T (2010) Risk factors for the evolutionary emergence of pathogens. J. R. Soc. Interface 7: 1455–74. 20410190 10.1098/rsif.2010.0123 PMC2935601

[28] Leigh GLJ (1970) Natural selection and mutability. Am. Nat. 104: 301–305.

[29] Harris TE (1963) The theory of branching processes. Dover phoenix editions.

[30] Wylie CS, Shakhnovich EI (2011) A biophysical protein folding model accounts for most mutational fitness effects in viruses. Proc. Natl. Acad. Sci. USA 108: 9916–9921. 10.1073/pnas.1017572108 PMC3116435 21610162

[31] Eyre-Walker A, Keightley PD (2007) The distribution of fitness effects of new mutations. Nat. rev. Genet. 8: 610–8. 10.1038/nrg2146 17637733

[32] Shih ACC, Hsiao TC, Ho MS, Li WH (2007) Simultaneous amino acid substitutions at antigenic sites drive influenza A hemagglutinin evolution. Proc. Natl. Acad. Sci. USA 104: 6283–8. 10.1073/pnas.0701396104 PMC1851070 17395716

[33] Lozovsky ER, Chookajorn T, Brown KM, Imwong M, Shaw PJ, et al (2009) Stepwise acquisition of pyrimethamine resistance in the malaria parasite. Proc. Natl. Acad. Sci. USA 106: 12025–30. 10.1073/pnas.0905922106 PMC2715478 19587242

[34] Chen HY, Di Mascio M, Perelson AS, Ho DD, Zhang L (2007) Determination of virus burst size in vivo using a single-cycle SIV in rhesus macaques. Proc. Natl. Acad. Sci. USA 104: 19079–84. 10.1073/pnas.0707449104 PMC2141911 18025463

[35] Keele BF, Giorgi EE, Salazar-Gonzalez JF, Decker JM, Pham KT, et al (2008) Identification and characterization of transmitted and early founder virus envelopes in primary HIV-1 infection. Proc. Natl. Acad. Sci. USA 105: 7552–7. 10.1073/pnas.0802203105 PMC2387184 18490657

[36] Wang GP, Sherrill-Mix SA, Chang KM, Quince C, Bushman FD (2010) Hepatitis C virus transmission bottlenecks analyzed by deep sequencing. J. Virol. 84: 6218–28. 10.1128/JVI.02271-09 PMC2876626 20375170

[37] Pearson JE, Krapivsky P, Perelson AS (2011) Stochastic Theory of Early Viral Infection: Continuous versus Burst Production of Virions. PLoS Comput. Biol. 7: e1001058. 10.1371/journal.pcbi.1001058 PMC3033366 21304934

[38] Loverdo C, Park M, Schreiber SJ, Lloyd-Smith JO (2012) Influence of viral replication mechanisms on within-host evolutionary dynamics. Evolution 66: 3462–3471. 23106710 10.1111/j.1558-5646.2012.01687.x

[39] Gavrilets S (1997) Evolution and speciation on holey adaptive landscapes. Trends Ecol. Evol 12: 307–12. 10.1016/S0169-5347(97)01098-7 21238086

[40] Sturtevant AH (1937) On the effects of selection on mutation rate. Q. Rev. Biol. 12: 464–467.

[41] Regoes R, Hamblin S, Tanaka M (2013) Viral mutation rates: modelling the roles of within-host viral dynamics and the trade-off between replication fidelity and speed. Proc. R. Soc. B 280: 20122047. 10.1098/rspb.2012.2047 PMC3574426 23135674

[42] Denamur E, Matic I (2006) Evolution of mutation rates in bacteria. Mol. Microbiol. 60: 820–7. 10.1111/j.1365-2958.2006.05150.x 16677295

[43] Kimura M (1967) On the evolutionary adjustment of spontaneous mutation rates. Genet Res 16: 39–42. 10.1007/BF00393983 4567209

[44] Gillespie JH (1981) Mutation Modification in a Random Environment. Evolution 35: 468–476. 28563590 10.1111/j.1558-5646.1981.tb04910.x

[45] Baer CF, Miyamoto MM, Denver DR (2007) Mutation rate variation in multicellular eukaryotes: causes and consequences. Nat. rev. Genet. 8: 619–31. 10.1038/nrg2158 17637734

