# Supplementary material for: Evolutionary Invasion and Escape in the Presence of Deleterious Mutations
Source: PLoS One. 2013 Jul 17;8(7):e68179. doi: 10.1371/journal.pone.0068179 (PMC3714272; doi:10.1371/journal.pone.0068179)
Supplement: File S1 — Calculations are detailed in the supporting pdf file, organized in the sections: • S1 General model. • S2 Different numbers of lethal mutations. • S3 Two steps towards a fitter mutant. • S4 Deleterious mutations. • S5 Within-host viral dynamics. • S6 Repetitively changing environment. (PDF) [file pone.0068179.s001.pdf]

Evolutionary invasion and escape in the presence of deleterious mutations

Claude Loverdo[1] and James O. Lloyd-Smith[1,2]

[1]Department of Ecology and Evolutionary Biology, University of California, Los Angeles, CA 90095, USA
[2]Fogarty International Center, National Institutes of Health, Bethesda, MD 20892, USA

Appendices

S1 General model

S1.1 The limit of small mutation rates

For a given mutation rate μ and initial strain 0, we define the probability to mutate to another strain k as $p_{0,k}(\mu)$. We divide the other strains, including lethal mutants, into immediate neighbors i that are one mutation away from strain 0, and all other neighbors j that are further away. We assume that the mutation rates at all sites are proportional to each other, so that in the limit of low mutation rates, $p_{0,i} = \mu r_{0,i}$ with $r_{0,i}$ a proportionality factor and $p_{0,j} = \Omega(\mu^2)$ or smaller; then we can define the probability that replication occurs without mutation, $p_{0,0} = 1 - \sum_i r_{0,i}\mu + \Omega(\mu^2) + ...$ The generating function starting from one replicator of strain 0 is:

$$g_0\left(\vec{z}\right) = \frac{1}{R_0+1} + \frac{R_0}{R_0+1} z_0 \left(p_{0,0} z_0 + \sum_i p_{0,i} z_i + \sum_j p_{0,j} z_j \right). \qquad (1)$$

Standard branching process theory implies that the probabilities of eventual extinction starting from one replicator of strain i e_i are the smallest positive solution of the system of equations $g_k(e_1, ..., e_n) = e_k$ (and the survival probability starting from one replicator of strain i is $s_i = 1 - e_i$) [1]. This leads to the following equation for the survival probabilities:

$$0 = s_0 - R_0(1 - s_0) \left(p_{0,0} s_0 + \sum_i p_{0,i} s_i + \sum_j p_{0,j} s_j \right). \qquad (2)$$

We differentiate with respect to μ:

$$\begin{aligned} 0 =&\partial_\mu s_0 + R_0 \partial_\mu s_0 \left(p_{0,0} s_0 + \sum_i p_{0,i} s_i + \sum_j p_{0,j} s_j \right) \\ &- R_0(1-s_0) \left(s_0 \partial_\mu p_{0,0} + \sum_i s_i \partial_\mu p_{0,i} + \sum_j s_j \partial_\mu p_{0,j} \right) \\ &- R_0(1-s_0) \left(p_{0,0} \partial_\mu s_0 + \sum_i p_{0,i} \partial_\mu s_i + \sum_j p_{0,j} \partial_\mu s_j \right) . \end{aligned} \tag{3}$$

We now evaluate the expression for $\mu = 0$. Without mutations, $p_{0,0}(\mu = 0) = 1$ and $p_{0,k\neq 0}(\mu = 0) = 0$. For immediate mutational neighbors, $\partial_\mu p_{0,i}|_{\mu=0} = r_{0,i}$. Because $p_{0,j} = \Omega(\mu^2)$ or smaller, therefore $\partial_\mu p_{0,j} = \Omega(\mu)$ or smaller, and so $\partial_\mu p_{0,j}|_{\mu=0} = 0$. Denoting $\widehat{x}$ as the value of x when $\mu = 0$,

$$0 = \widehat{\partial_\mu s_0} + R_0 \widehat{\partial_\mu s_0} \widehat{s_0} - R_0(1-\widehat{s_0}) \left(-\sum_i r_{0,i} \widehat{s_0} + \sum_i r_{0,i} \widehat{s_i} \right) - R_0(1-\widehat{s_0}) \widehat{\partial_\mu s_0}. \tag{4}$$

This can be simplified to yield:

$$\widehat{\partial_\mu s_0} = \frac{R_0(1-\widehat{s_0})}{1 - R_0 + 2R_0 \widehat{s_0}} \sum_i r_{0,i} (\widehat{s_i} - \widehat{s_0}). \tag{5}$$

If $R_0 < 1$, $\widehat{s_0} = 0$, and:

$$s_0 = \mu \frac{R_0}{1 - R_0} \sum_i r_{0,i} \widehat{s_i} + \Omega(\mu^2). \tag{6}$$

If $R_0 > 1$, $\widehat{s_0} = 1 - 1/R_0$, and:

$$s_0 = 1 - \frac{1}{R_0} + \frac{\mu}{R_0 - 1} \sum_i r_{0,i} (\widehat{s_i} - \widehat{s_0}) + \Omega(\mu^2). \tag{7}$$

The sign of this derivative summarizes whether a small amount of mutation leads to a higher survival probability than no mutation. In both cases, this sign is determined by the sign of $\sum_i r_{0,i}(\widehat{s_i} - \widehat{s_0})$, i.e. mutations are beneficial if the weighted average of the survival probabilities of the neighboring mutants in the absence of mutations is larger than the survival probability of the initial strain without mutations. This criterion is sufficient for the mutations to be beneficial.

S1.2 Approximations for lethal mutations

One can notice that in equation (1) of the main text, the probability that there is no lethal mutation is $(1 - \mu)^L$. If the mean number of lethal mutations per replication is U (μL in our notation), then often in the literature (see for example [2]) the probability that there is no lethal mutation is taken as $\exp(-U)$. This is the probability to have zero mutations for a Poissonian distribution, implying that there can be any number of lethal mutations, whereas in our model there could be at most L lethal mutations. However, in practice, for μ small, $(1 - \mu)^L \simeq \exp(-\mu L)$, and this approximation is sometimes used in the following calculations.

S1.3 Iterative approximations for survival probabilities

The survival probabilities are the solutions of:

$$0 = s_1 - R_1(1-s_1)(1-\mu)^L((1-\mu)s_1 + \mu s_2) \tag{8}$$

and the analogous expression for $1 \leftrightarrow 2$ (i.e. interchanging the indices 1 and 2 in the equation). We derive approximate solutions by iteration, including successively more steps of mutation.

The first step is the survival probability with only lethal mutations, solution of $0 = s_i - R_i(1-s_i)s_i(1-\mu)^L$:

$$s_i^{(0)} = \max\left\{0, \frac{R_i(1-\mu)^L - 1}{R_i(1-\mu)^L}\right\}. \tag{9}$$

If we replace s_2 by $s_2^{(0)}$ in the analogous of equation (8) for $1 \leftrightarrow 2$, this equation is then a quadratic equation for s_1, that we can solve to obtain $s_1^{(1)}$ as a function of $s_2^{(0)}$, using the additional property that survival probabilities are positive. Recursively, the higher-order solutions follow with:

$$s_i^{(k+1)} = \frac{1}{2R_i(1-\mu)^{L+1}}\left(\alpha_i + \sqrt{\alpha_i^2 + 4(1-\mu)^{2L+1}\mu R_i^2 s_j^{(k)}}\right), \tag{10}$$

with $\alpha_i = -1 + R_i(1-\mu)^L(1-\mu-\mu s_j^{(k)})$, with $(i,j) = (1,2)$ or $(2,1)$.

S1.4 Further approximations in the regime of evolutionary escape ($R_1 < 1$, $R_2 > 1$)

When $R_1 < 1$, $R_1(1-\mu)^L < 1$, thus $s_1^{(0)} = 0$, leading to $s_2^{(1)} = \max\left\{0, 1 - 1/(R_2(1-\mu)^{L+1})\right\}$. We define $\widetilde{s_2}^{(1)} = 1 - 1/(R_2(1-\mu)^{L+1})$. This expression makes intuitive sense, because in this parameter regime, the strain 1 is similar to an additional lethal mutant neighbor for strain 2, so $s_2^{(1)}$ is like $s_2^{(0)}$ but with $L+1$ instead of L. When μ is close to μ^{opt}, it can be shown that $R_2(1-\mu)^{L+1} > 1$, thus $s_2^{(1)} = \widetilde{s_2}^{(1)}$. This leads to:

$$s_1 \simeq \frac{1}{2R_1(1-\mu)^{L+1}}\left(\widetilde{\alpha_1} + \sqrt{\widetilde{\alpha_1}^2 + 4(1-\mu)^{2L+1}\mu R_1^2 \widetilde{s_2}^{(1)}}\right), \tag{11}$$

with $\widetilde{\alpha_1} = -1 + R_1(1-\mu)^L(1-\mu-\mu\widetilde{s_2}^{(1)})$.

Then, we assume $\mu\widetilde{s_2}^{(1)} \ll 1 - R_1(1-\mu)^{L+1}$, leading to a general approximation for s_1:

$$s_1 \simeq \frac{R_1\mu(R_2(1-\mu)^{L+1} - 1)}{R_2(1-\mu)(1 - R_1(1-\mu)^{L+1})}. \tag{12}$$

We can now optimize the approximate survival probability to learn about μ^{opt}. The derivative of (12) with respect to μ equals zero when:

$$0 = \left(1 - R_1(1-\mu)^{L+1}\right)\left(1 - R_2(1-\mu)^{L+1}\right) + \mu(L+1)(1-\mu)^{L+1}(R_2 - R_1). \tag{13}$$

We further assume that $\mu \ll 1$, leading to $(1-\mu)^{L+1} = \exp(\ln(1-\mu)(L+1)) \simeq \exp(-\mu(L+1))$. The equation to solve is

$$0 = \left(1 - R_1 e^{-X}\right)\left(1 - R_2 e^{-X}\right) + X e^{-X}(R_2 - R_1), \tag{14}$$

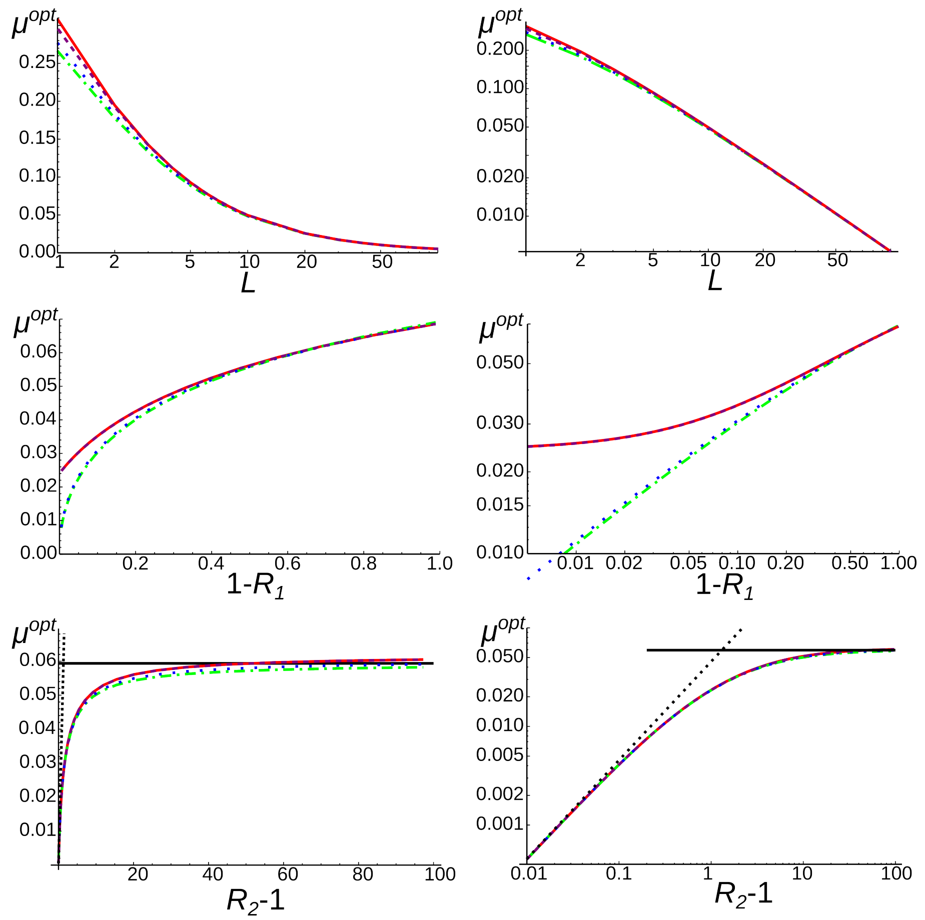

Figure S1: Dependence of the optimal mutation rate μ^{opt} on the number of lethal mutations and the fitnesses of the two viable strains, and comparison of the approximations. The two columns show the same data with linear scale on the left and log scale on the right (except for the top left panel where only the y-axis is linear). Different solutions are denoted with different line styles, as follows: exact solution (red solid line), from $\widetilde{s_1}^{(2)}$ (purple dashed line), with a development in $\mu\widetilde{s_2}^{(1)}$ (13) (blue dotted line), and with the additional $(1-\mu)^{L+1} \simeq \exp(-\mu(L+1))$ (14) (green dot-dashed line). The bottom row shows two additional approximations: the development in $R_2 \to 1^+$ (15) (black dotted line), and the development in R_2 large (16) (black solid line). Unless indicated otherwise, $L = 10$, $R_1 = 2/3$ and $R_2 = 9$.

with $X = \mu(L+1)$. In this regime, as the equation to solve is function of X and not μ and L independently, $\mu^{opt} \propto 1/(L+1)$. As expected, the more numerous the lethal mutants, the riskier mutations become, and the smaller the optimal mutation rate.

Regime $\mathbf{R_2 - 1 \ll 1}$ In the limit $R_2 = 1+\epsilon$, $\mu(L+1) = \Omega(\epsilon)$, and (14) leads to:

$$\mu^{opt} \to \frac{R_2 - 1}{2(L+1)}. \quad (15)$$

Regime $\mathbf{R_2 \gg 1}$ In the limit R_2 large, (14) can be simplified as:

$$X = 1 - R_1 e^{-X}. \quad (16)$$

This equation can only be solved numerically, but importantly it shows that the result does not depend on R_2.

Comparison of the approximations Figure S1 shows the comparison of the approximations with the exact solution. $\widetilde{s_1}^{(2)}$ gives a good approximation except for L small. The development in $\mu\widetilde{s_2}^{(1)}$ (13) and the further step $(1-\mu)^{L+1} \simeq \exp(-\mu(L+1))$ (14) fit well provided $1 - R_1$ is not too close to 0. The bottom panels show that the solutions of (15) and (16) are good approximations in their intended regimes.

S2 Different numbers of lethal mutations

Here we discuss how μ^{opt} is influenced by different values of L_1 and L_2, restricting the analysis to the regime where $R_1 < 1$ and $R_2 > 1$.

General equations are the same as for the simple model, except that strain i has L_i lethal neighbors. By analogy with (10), our starting point is:

$$s_1^{(2)} = \frac{1}{2}\left(1 - \frac{1}{R_1(1-\mu)^{L_1+1}} - \frac{\mu s_2^{(1)}}{1-\mu} + \sqrt{\frac{4\mu s_2^{(1)}}{1-\mu} + \left(1 - \frac{1}{R_1(1-\mu)^{L_1+1}} - \frac{\mu s_2^{(1)}}{1-\mu}\right)^2}\right), \quad (17)$$

with $s_2^{(1)} = 1 - 1/(R_2(1-\mu)^{L_2+1})$.

When $1 - R_1$ is not very small compared to 1, $\mu s_2^{(1)}$ is small compared to the other terms, and the development of $s_1^{(2)}$ leads to:

$$s_1 \simeq \frac{R_1\mu(1-\mu)^{L_1}}{1 - R_1(1-\mu)^{L_1+1}} \frac{R_2(1-\mu)^{L_2+1} - 1}{R_2(1-\mu)^{L_2+1}}. \quad (18)$$

which differentiated with respect to μ leads to the following equations for the optimal mutation rate:

$$0 = 1 + (L_2 - L_1)\mu - R_1(1+\mu(L_2+1))(1-\mu)^{L_1+1} - R_2(1-\mu(L_1+1))(1-\mu)^{L_2+1} + R_1R_2(1-\mu)^{L_1+L_2+2}. \quad (19)$$

We confirm that if $L_1 = L_2 = L$, we recover (13).

As for the simple model, we can approximate $(1-\mu)^{L_i+1}$ by $\exp(-\mu(L_i+1))$ in the limit $L_i \gg 1$. Once again we set $X_i = \mu(L_i+1)$, yielding the equation to solve for μ^{opt}:

$$1 + X_2 - X_1 - R_1(1+X_2)e^{-X_1} - R_2(1-X_1)e^{-X_2} + R_1R_2e^{-(X_1+X_2)} = 0. \quad (20)$$

In the limit $L_1 \gg L_2$, we have $X_1 \gg X_2$ and so (20) simplifies to $1 - X_1 - R_1e^{-X_1} \simeq 0$. Similarly in the limit $L_2 \gg L_1$, (20) simplifies to $1 + X_2 - R_2e^{-X_2} \simeq$

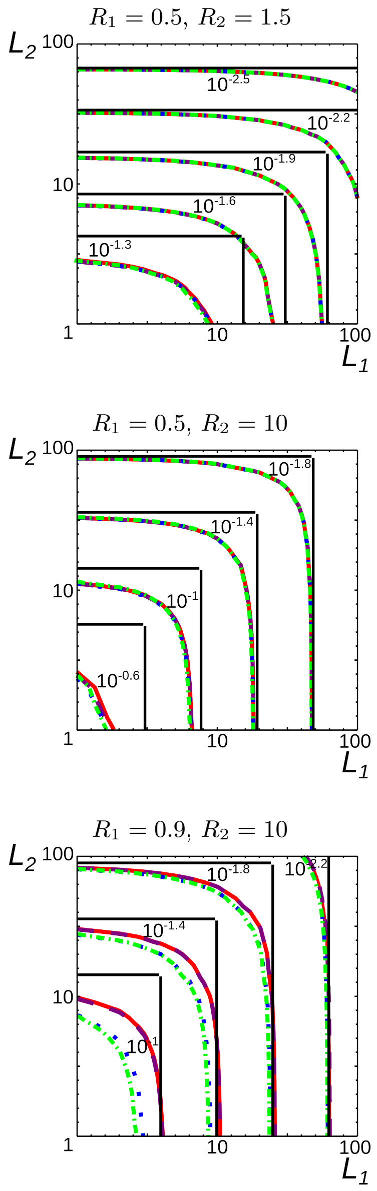

Figure S2: Value of μ^{opt} as a function of L_1 and L_2 (y axis), for the exact solution (solid red lines) and approximations $s_1^{(2)}$ (17) (purple dashed lines), development in $\mu s_2^{(1)}$ small (19) (blue dotted lines), further approximation of L_1 and L_2 large (20) (green dot-dashed lines), and more extreme approximation (21) (black straight lines).

0. Therefore, in the regimes where one strain is threatened by many more lethal mutations than the other, it is the optimization on this strain which determines μ^{opt}, independent of the parameters of the less threatened strain.

As an extreme approximation, we can consider the optimal mutation rate as

a piecewise combination of these two cases:

$$\mu^{opt} \simeq \min \left\{ \left(\frac{X_1}{L_1+1} \middle| 1 - X_1 - R_1 e^{-X_1} = 0 \right), \left(\frac{X_2}{L_2+1} \middle| 1 + X_2 - R_2 e^{-X_2} = 0 \right) \right\}. \tag{21}$$

Figure S2 shows how the exact numerical solution for μ^{opt} compares to a series of approximations, as a function of L_1 and L_2. Values of μ^{opt} calculated numerically from $s_1^{(2)}$ (17) work well except when L_1 and L_2 are both very small; those calculated from the approximations for $\mu s_2^{(1)}$ small, (19) and (20), work well if $1 - R_1$ is not too small. The rough approximation (21) works remarkably well in the limit L_1 and L_2 large, especially if one of them is much larger than the other.

S3 Two steps towards a fitter mutant

In the case of two binary loci, described in the text:

$$g_{00} = \frac{1}{1+R_{00}} + \frac{R_{00}}{1+R_{00}} \left(1 - (1-\mu)^L + (1-\mu)^L \left((1-\mu)^2 z_{00} + \mu(1-\mu) z_{01} + \mu(1-\mu) z_{10} + \mu^2 z_{11}\right)\right) \tag{22}$$

leading to the equation for the survival probability:

$$s_{00} = R_{00}(1-s_{00})(1-\mu)^L((1-\mu)^2 s_{00} + \mu(1-\mu)s_{01} + \mu(1-\mu)s_{10} + \mu^2 s_{11}) \tag{23}$$

and analogous equations for the other strains.

Our analysis focuses on the case where both sites must mutate in order for the virus to attain a higher fitness (sometimes called a “jackpot model”). That is, we take $R_{00} = R_{01} = R_{10} = R_1$ and $R_{11} = R_2$. In this case we have the set of equations:

$$s_0 = R_1(1-s_0)(1-\mu)^L((1-\mu)^2 s_0 + 2\mu(1-\mu)s_1 + \mu^2 s_2), \tag{24}$$

$$s_1 = R_1(1-s_1)(1-\mu)^L(((1-\mu)^2 + \mu^2)s_1 + \mu(1-\mu)s_0 + \mu(1-\mu)s_2) \text{ and} \tag{25}$$

$$s_2 = R_2(1-s_2)(1-\mu)^L((1-\mu)^2 s_2 + 2\mu(1-\mu)s_1 + \mu^2 s_0). \tag{26}$$

These equations are solved numerically to build figure 4 of main text.

Analogous calculations and conclusions can be made when more mutations are needed, and show the generality of the qualitative findings from the 2-step case (figure S3).

S4 Deleterious mutations

When all deleterious mutations are strictly lethal When all deleterious mutations are strictly lethal, the survival probabilities are found from the system of equations:

$$s_1 = R_1(1-s_1)(1-\mu)^L((1-\mu)s_1 + \mu s_2), \tag{27}$$

and the symmetric equation for $1 \leftrightarrow 2$.

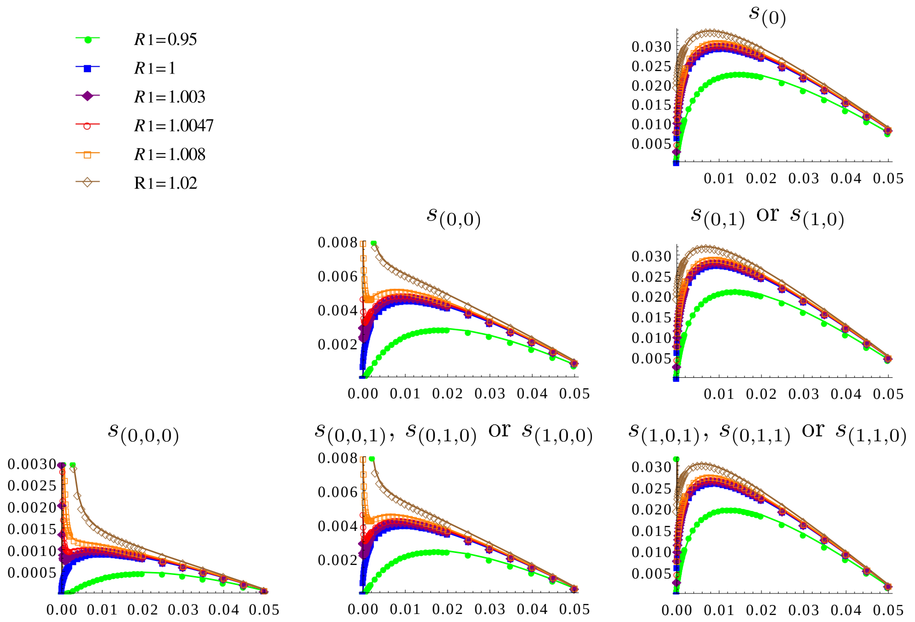

Figure S3: Survival probability as a function of the mutation rate, when one, two or three sites must mutate in order to increase fitness (i.e. a jackpot-like landscape, with fitness R_1 for all strains except the adapted one which has R_2). The three rows of panels show the results for a 1-step, 2-step and 3-step path to adaptation, from top to bottom. $R_2 = 9$, $L = 10$.

When one deleterious mutation leads to a reduced fitness R_d, and two or more deleterious mutations are lethal In the case when one deleterious mutation leads to a reduced fitness R_d, and two or more deleterious mutations to $R_0 = 0$ (lethal), the system is:

$$s_1 = R_1(1-s_1)(1-\mu)^{L-1}((1-\mu)^2 s_1 + (1-\mu)\mu s_2 + L\mu(1-\mu)s_{1d} + L\mu^2 s_{2d}), \quad (28)$$

$$s_{1d} = R_d(1-s_{1d})(1-\mu)^{L-1}((1-\mu)^2 s_{1d} + (1-\mu)\mu s_{2d} + \mu(1-\mu)s_1 + \mu^2 s_2), \quad (29)$$

and symmetric equations for $1 \leftrightarrow 2$.

When any non-zero number of deleterious mutations leads to a reduced fitness R_d, or when deleterious effects are multiplicative For the two other cases shown in figure 5 of the article, with $s_{j,i}$ the survival probability starting from a replicator with allele j at the adaptive site and i mutated deleterious sites, the system is:

$$s_{1,i} = R_{1,i}(1-s_{1,i}) \sum_{p=0}^{i} \binom{i}{p} \sum_{q=0}^{L-i} \binom{L-i}{q} \mu^{p+q}(1-\mu)^{L-p-q}((1-\mu)s_{1,i+q-p} + \mu s_{2,i+q-p}), \quad (30)$$

and symmetric equations for $1 \leftrightarrow 2$, with $s_{j,0} = R_j$. For the case of a uniform deleterious effect, we set $R_{j,i>0} = R_d$, and for the case of multiplicative effects we set $R_{j,i} = R_j \alpha^i$.

S5 Within-host viral dynamics

S5.1 Generating function

With probability $1 - q_i$, a virion of strain i does not successfully infect a host cell (term in $(1 - q_i)z_i^0$ in the generating function). When a cell is successfully infected (probability q_i), it generates offspring virions according to a geometric distribution with mean N_i (generating function $1/(1 + N_i(1 - z))$), each of which can be independently mutated ($z = (1 - (1 - \mu)^L)z_i^0 z_j^0$ (lethal mutants) $+(1 - \mu)^{L+1} z_i$ (no mutation) $+(1 - \mu)^L \mu z_j$ (mutation at the adaptive site but no lethal mutation)). The generating function starting from one virion of strain 1 is then:

$$g_1(z_1, z_2) = 1 - q_1 + \frac{q_1}{1 + N_1(1 - \mu)^L(1 - (1 - \mu)z_1 - \mu z_2)}. \tag{31}$$

The corresponding equations for the survival probabilities are:

$$0 = s_1 - (q_1 - s_1)N_1(1 - \mu)^L((1 - \mu)s_1 + \mu s_2), \tag{32}$$

and the analogue with $1 \leftrightarrow 2$.

The iterative approximations start with:

$$s_i^{(0)} = \max\left\{0, q_i\left(1 - \frac{1}{R_i(1 - \mu)^L}\right)\right\} \tag{33}$$

and proceed according to:

$$s_i^{(k+1)} = \frac{1}{2}\left(q_i - \frac{1}{N_i(1 - \mu)^{L+1}} - \frac{\mu s_j^{(k)}}{1 - \mu} + \sqrt{\frac{4\mu q_i s_j^{(k)}}{1 - \mu} + \left(q_i - \frac{1}{N_i(1 - \mu)^{L+1}} - \frac{\mu s_j^{(k)}}{1 - \mu}\right)^2}\right). \tag{34}$$

S5.2 No change in the dependence of survival probability on mutation rate when q_i and N_i are adjusted with R_i constant

Let us define $s_i = s_i(q_1, N_1, q_2, N_2)$, $s_i' = s_i(rq_1, N_1/r, rq_2, N_2/r)$ and $\widetilde{s_i} = s_i'/r$. Using equation (32),

$$0 = s_1' - (rq_1 - s_1')\frac{N_1}{r}(1 - \mu)^L((1 - \mu)s_1' + \mu s_2'), \tag{35}$$

which can be rewritten as:

$$0 = r\widetilde{s_1} - r(q_1 - \widetilde{s_1})N_1(1 - \mu)^L((1 - \mu)\widetilde{s_1} + \mu\widetilde{s_2}). \tag{36}$$

Thus $\widetilde{s_i}$ and s_i both satisfy the equations for survival probabilities for this system. Because there is at most one solution of these equations in $(0, 1] \times (0, 1]$ [1], we have shown that $s_i(rq_1, N_1/r, rq_2, N_2/r) = rs_i(q_1, N_1, q_2, N_2)$.

S5.3 Approximations in the regime of evolutionary escape ($R_1 < 1$, $R_2 > 1$)

We use $s_1^{(2)}$ as an approximation, with $s_2^{(1)} = q_2(1 - 1/(R_2(1 - \mu)^{L+1}))$. When studying the optimum numerically, we observe that there are two regimes: $q_1 > q_2$ and $q_1 \ll q_2$.

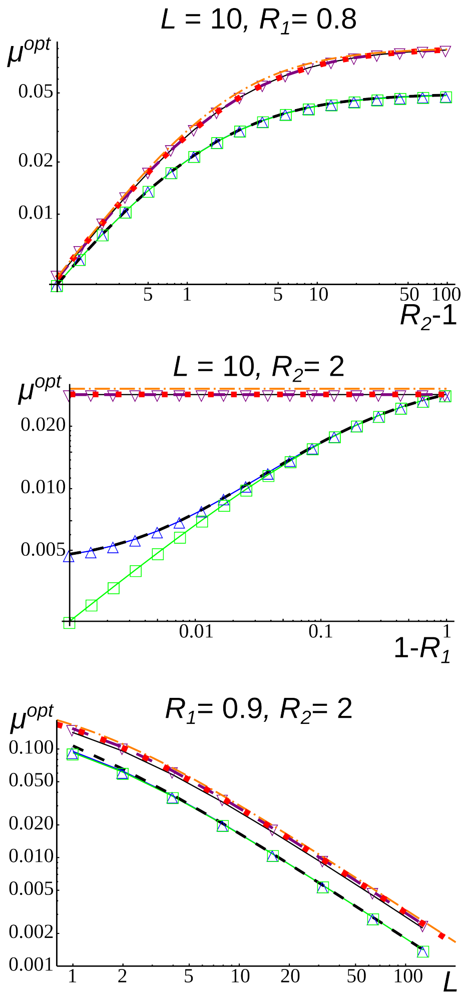

Figure S4: The optimal mutation rate for the model of within-host viral infection as a function of $R_2 - 1$, $1 - R_1$ and L, comparing the approximations in the two parameter regimes. Each subpanel shows results for two sets of parameters q_1 and q_2. In the regime where $q_1 > q_2$ ($q_1 = 10^{-3}, q_2 = 10^{-4}$), the exact solution (black dashed line) is represented along with the approximations $s_1^{(2)}$ (34) (blue up triangles and blue solid line) and approximation $\mu s_2^{(1)}$ small (13) (green squares and green solid line). In the regime where $q_1 \ll q_2$ ($q_1 = 10^{-5}, q_2 = 10^{-1}$), the exact solution (black solid line) is represented along with the approximations $s_1^{(2)}$ (34) (purple down triangles and purple dashed line), q_1 small (40) (red dotted line), and the further approximation (41) (orange dot-dashed line).

Regime $\mathbf{q_1 > q_2}$ We can proceed as in the simple model: when $1 - R_1$ is not too small, we develop $s_1^{(2)}$ assuming $\mu s_2^{(1)}$ small (for fixed R_2, $s_2^{(1)}$ scales as q_2, so the smaller q_2, the better this approximation), and obtain:

$$s_1 \simeq \frac{(1-\mu)^L \mu N_1 q_1 s_2^{(1)}}{1 - (1-\mu)^{L+1} N_1 q_1} = \frac{(1-\mu)^L \mu R_1 q_2}{1 - (1-\mu)^{L+1} R_1} \left(1 - \frac{1}{R_2 (1-\mu)^{L+1}}\right). \quad (37)$$

When differentiated with respect to μ, it leads to the following equation for μ^{opt}:

$$0 = \left(1 - R_1(1-\mu)^{L+1}\right)\left(1 - R_2(1-\mu)^{L+1}\right) + \mu(L+1)(1-\mu)^{L+1}(R_2 - R_1), \tag{38}$$

which is the same as (13) for the simple model. Therefore the conclusions are the same as for the simple model.

Regime $\mathbf{q_1 \ll q_2}$ Developing $s_1^{(2)}$ in the limit q_1 small, we obtain:

$$s_1 \simeq q_1 \left(1 + \frac{R_2(1-\mu)q_1}{\mu q_2 R_1 \left(1 - (1-\mu)^{L+1} R_2\right)}\right), \tag{39}$$

which differentiated with respect to μ is proportional to:

$$-1 + (1-\mu)^{L+1} R_2 (1 - (L+1)\mu). \tag{40}$$

By assuming that this expression equals zero when $\mu = \mu^{opt}$, we obtain a good approximation (figure S4). If $R_2 - 1 \ll 1$, then $\mu L \ll 1$, thus (40) leads to $\mu^{opt} \simeq (R_2 - 1)/(2(L+1))$. In the limit $R_2 \gg 1$, (40) leads to $\mu^{opt} \simeq 1/(L+1)$. These limiting expressions can be combined in:

$$\mu^{opt} \simeq \frac{R_2 - 1}{(L+1)(R_2+1)}, \tag{41}$$

which, though only rigorous for $R_2 - 1 \ll 1$ and $R_2 \gg 1$ is a good approximation for most of the R_2 range (figure S4).

S6 Repetitively changing environment

Achieving a low mutation rate can be costly (resources used to maintain repair mechanisms, replication slowed by proofreading steps, etc.) [3, 4, 5, 6]. In the following discussion, we completely neglect this aspect, focus only on the impact of the mutations on fitness, how the interplay between adaptive mutations and deleterious load affects survival. Adding a cost to fidelity would increase the optimal mutation rate.

We study evolutionary invasion and escape, which is adaptation to environmental change. In the main text, we focus on one step of environmental change. In this section we discuss the case of several successive environmental changes.

There are three different relevant time-scales: the time τ_e between 2 environmental changes, the time τ_a to adapt via mutations to an environmental change, and the time τ_m for the mutation rate to change. It is possible to have $\tau_m \gg \tau_a$, for example when one or a few mutations are needed to adapt to the environment whereas many mutations are needed to modify the mutation rate. Then there are three situations:

- If $\tau_e \ll \tau_a$, then environmental changes are too rapid for genetic changes to be a relevant response.
- If $\tau_e \gg \tau_m$, then mutation rates are selected to be low when the environment is stable; when the environment changes, mutants with higher mutation rates are most likely to produce adaptive mutations, and will hitch-hike to high frequency with these mutations, but will decline again when the environment stabilizes [7].

- If $\tau_a \ll \tau_e \ll \tau_m$, the mutation rate will evolve towards an optimum between limiting the deleterious load but allowing for adaptation when the environment changes. We focus on this regime.

Let us assume that replicators face successive environments $I = 1, 2, 3,$... In environment I, the best reproducing genotype is i. Let us assume that τ_e is long enough that before a new environmental change, the population is limited in its growth (for example by resource availability) and has reached the mutation-selection balance. The proportion $p_{j,I-1}$ of replicators j in environment $I-1$ is of the order of one for the replicator $j = i-1$ (and decreases for increasing mutation rates), and of the order of μ or smaller for all the other replicators (and increasing with the mutation rate). Let us assume that an average of N_I replicators are passed from the environment $I-1$ to I. N_I could be a constant, for example a fixed carrying capacity for environment $I-1$, or the size of a founder population that has migrated from environment $I-1$ to environment I. If N_I is constant, $dN_I/d\mu = 0$. If N_I depends on the replicator fitness, then at the mutation-selection balance, N_I decreases with increasing mutation rates, i.e. $dN_I/d\mu < 0$. Let us assume that the number of replicators passed to the next environment follows a Poisson distribution. We define $s_{j,I}$ as the probability of survival of a lineage initiated by one replicator of strain j in environment I.

The survival probability through all the environmental changes is $s = \prod_I s_I$, with s_I the survival probability for one step of environmental change from $I-1$ to I. If there are k_j replicators of strain j passed to the new environment I, the survival probability is $1 - \prod_j (1 - s_{j,I})^{k_j}$. We assume that the survival probabilities of all replicators are independent, and consequently the average s_I is:

$$s_I = 1 - \prod_j \sum_{k_j} (1 - s_{j,I})^{k_j} \frac{(N_I p_{j,I-1})^{k_j}}{k_j!} \exp(-N_I p_{j,I-1}) = 1 - \prod_j \exp(-N_I p_{j,I-1} s_{j,I}). \tag{42}$$

To infer which effects are predominant, we study how s varies with the mutation rate:

$$\frac{ds}{d\mu} = s \sum_I \frac{1}{s_I} \frac{ds_I}{d\mu}, \tag{43}$$

$$\frac{ds}{d\mu} = s \sum_I \frac{\prod_j \exp(-N_I p_{j,I-1} s_{j,I})}{1 - \prod_j \exp(-N_I p_{j,I-1} s_{j,I})} \sum_j \left(\frac{dN_I}{d\mu} p_{j,I-1} s_{j,I} + N_I \frac{dp_{j,I-1}}{d\mu} s_{j,I} + N_I p_{j,I-1} \frac{ds_{j,I}}{d\mu} \right), \tag{44}$$

$$\frac{ds}{d\mu} = s \sum_I \frac{(1 - s_I)}{s_I} \sum_j \left(\frac{dN_I}{d\mu} p_{j,I-1} s_{j,I} + N_I \frac{dp_{j,I-1}}{d\mu} s_{j,I} + N_I p_{j,I-1} \frac{ds_{j,I}}{d\mu} \right). \tag{45}$$

Because of the factor $(1 - s_I)/s_I$, the steps that will matter more are the steps with the smallest survival probability, which are the limiting steps.

We discuss below which factors are the most important for a given step of environmental change. There are many possible scenarios for N_I. If N_I depends on the fitnesses of the population of replicators in environment $I-1$, $dN_I/d\mu < 0$, and lower mutation rates are favored. We will focus on the case where N_I does not depend on the overall fitness, or weakly, so that we can

neglect the terms proportional to $dN_I/d\mu$. In this scenario, we only need to compare the terms $s_{j,I}dp_{j,I-1}/d\mu$ and $p_{j,I-1}ds_{j,I}/d\mu$.

- For any $j \neq i$, $ds_{j,I}/d\mu$ is expected to depend strongly on μ, except for specific cases. Besides, $p_{j\neq(i-1),I-1} \ll p_{i-1,I-1}$. Consequently, for any $j \neq i$ or $i-1$, it is likely that $p_{j,I-1}ds_{j,I}/d\mu$ is much smaller than $p_{i-1,I-1}ds_{i-1,I}/d\mu$.

- We expect $ds_{i,I}/d\mu$ to be small, and as $p_{i,I-1} \ll p_{i-1,I-1}$, then it is likely that $p_{i,I-1}ds_{i,I}/d\mu$ is much smaller than $p_{i-1,I-1}ds_{i-1,I}/d\mu$.

- As the replicator of strain $i-1$ is the most abundant in environment $I-1$, $dp_{i-1,I-1}/d\mu \simeq -\sum_{j\neq(i-1)} dp_{j,I-1}/d\mu$. Thus $\sum_j s_{j,I}\frac{dp_{j,I-1}}{d\mu} \simeq \sum_{j\neq(i-1)}(s_{j,I} - s_{i-1,I})\frac{dp_{j,I-1}}{d\mu}$. As i is the fittest strain in environment I, $s_{j\neq i,I} < s_{i,I}$. In most cases, $s_{j\neq i,I} \ll s_{i,I}$, which guarantees $(s_{i,I} - s_{i-1,I})\frac{dp_{i,I-1}}{d\mu}$ to be the most important of the sum. This term is positive: when the mutation rate increases, there are more pre-existing mutants i in the environment $I-1$ which will be adaptive to the new environment I.

Thus the two terms that are the most likely to be significant are $p_{i-1,I-1}ds_{i-1,I}/d\mu$ and $(s_{i,I}-s_{i-1,I})\frac{dp_{i,I-1}}{d\mu}$. $p_{i-1,I-1}$ is of the order of one minus corrections proportional to the mutation rate. If all mutation rates are similar, $\frac{dp_{i,I-1}}{d\mu}$ is also about one. For values of μ for which $s_{i-1,I}$ varies strongly, $p_{i-1,I-1}ds_{i-1,I}/d\mu$ will dominate. In the article, we maximize the survival probability analogous to $s_{i-1,I}$. Where μ is close to $\mu^{opt,1step}$ as calculated for one step, $(s_{i,I} - s_{i-1,I})\frac{dp_{i,I-1}}{d\mu}$ will be important, and overall will shift the rate maximizing survival to values somewhat larger than $\mu^{opt,1step}$.

To summarize, successive environmental changes (occurring faster than the time required for the mutation rate to evolve) will select for a mutation rate close to the optimal mutation rate we have calculated for one step (taking the step with the smallest survival probability, with the strain most adapted to previous environment as the initial replicator). A more detailed model is necessary to calculate corrections, due to the number of replicators passed from the previous environment (which decreases the optimal mutation rate) and the number of pre-existing mutants (which acts in the opposite way).

References

[1] Harris TE. The theory of branching processes. Dover phoenix editions; 1963.

[2] Bull JJ. 2008. The optimal burst of mutation to create a phenotype. *J. Theor. Biol.*. **254**,667–73.

[3] Sniegowski PD, Gerrish PJ, Johnson T, Shaver A. 2000. The evolution of mutation rates: separating causes from consequences. *BioEssays.* **22**,1057–66.

[4] Furió V, Moya A, Sanjuán R. 2005. The cost of replication fidelity in an RNA virus. *Proc. Natl. Acad. Sci. USA.* **102**,10233–7.

[5] Thébaud G, Chadoeuf J, Morelli MJ, McCauley JW, Haydon DT. 2010. The relationship between mutation frequency and replication strategy in positive-sense single-stranded RNA viruses. *Proc. R. Soc. B.* **277**,809–17.

[6] Regoes R, Hamblin S, Tanaka M. 2013. Viral mutation rates: modelling the roles of within-host viral dynamics and the trade-off between replication fidelity and speed. *Proc. R. Soc. B.* **280**,20122047.

[7] Denamur E, Matic I. 2006. Evolution of mutation rates in bacteria. *Mol. Microbiol.* **60**,820–7.
